# Retraction Note: PI3K/Akt pathway and Nanog maintain cancer stem cells in sarcomas

**DOI:** 10.1038/s41389-024-00519-0

**Published:** 2024-05-29

**Authors:** Changhwan Yoon, Jun Lu, Brendan C. Yi, Kevin K. Chang, M. Celeste Simon, Sandra Ryeom, Sam S. Yoon

**Affiliations:** 1 Department of Surgery, Memorial Sloan Kettering Cancer Center, New York, NY USA; 2 Department of Gastric Surgery, Fujian Medical University Union Hospital, Fujian, China; 3 Abramson Family Cancer Research Institute, Perelman School of Medicine, University of Pennsylvania, Philadelphia, PA 19104 USA; 4 Department of Cancer Biology, Perelman School of Medicine, University of Pennsylvania, Philadelphia, PA 19104 USA

Retraction to: *Oncogenesis* 10.1038/s41389-020-00300-z, published online 19 January 2021

The authors have retracted this article. After publication, concerns were raised regarding the data presented in the figures. Specifically:

- In Fig. 4C, the sh.Scr + DMSO tumor image appears highly similar to the Fig. 6A upper tumor image of [1]
- In Fig. 4C, the sh.Nanog + Dox tumor image appears highly similar to the Fig. 6B Cisplatin tumor image of [2]
- In Fig. 4D, the Cleaved Caspase-3 sh.Scr Dox image has elements that appear highly similar to the Fig. 6D sh.AKT1/2 -Dox image
- In Fig. 6C, the sh.AKT1/2 + Dox tumor image appears highly similar to the Fig. 6B Fasudil tumor image of [2]

Authors Changhwan Yoon, Kevin K. Chang, M. Celeste Simon, Sandra Ryeom, and Sam S. Yoon agree with this retraction. Authors Jun Lu and Brendan C. Yi have not responded to correspondence regarding this retraction.
